# Internet-based Surveillance Systems and Infectious Diseases Prediction: An Updated Review of the Last 10 Years and Lessons from the COVID-19 Pandemic

**DOI:** 10.1007/s44197-024-00272-y

**Published:** 2024-08-14

**Authors:** Hannah McClymont, Stephen B. Lambert, Ian Barr, Sotiris Vardoulakis, Hilary Bambrick, Wenbiao Hu

**Affiliations:** 1 Ecosystem Change and Population Health (ECAPH) Research Group, School of Public Health and Social Work, Queensland University of Technology (QUT), Brisbane, Australia; 2 Communicable Diseases Branch, Queensland Health, Brisbane, Australia; 3 National Centre for Immunisation Research and Surveillance, Sydney Children’s Hospitals Network, Westmead, Australia; 4 WHO Collaborating Centre for Reference and Research on Influenza, The Peter Doherty Institute for Infection and Immunity, Melbourne, Australia; 5 Department of Microbiology and Immunology, University of Melbourne, Melbourne, Australia; 6 Health Research Institute, University of Canberra, Canberra, Australia; 7 National Centre for Epidemiology and Population Health, College of Health and Medicine, The Australian National University, Canberra, Australia; 8 Healthy Environments and Lives (HEAL) National Research Network, Canberra, Australia

**Keywords:** Early Warning Systems, Internet-based Surveillance, COVID-19, Influenza, Dengue, mHealth

## Abstract

The last decade has seen major advances and growth in internet-based surveillance for infectious diseases through advanced computational capacity, growing adoption of smart devices, increased availability of Artificial Intelligence (AI), alongside environmental pressures including climate and land use change contributing to increased threat and spread of pandemics and emerging infectious diseases. With the increasing burden of infectious diseases and the COVID-19 pandemic, the need for developing novel technologies and integrating internet-based data approaches to improving infectious disease surveillance is greater than ever. In this systematic review, we searched the scientific literature for research on internet-based or digital surveillance for influenza, dengue fever and COVID-19 from 2013 to 2023. We have provided an overview of recent internet-based surveillance research for emerging infectious diseases (EID), describing changes in the digital landscape, with recommendations for future research directed at public health policymakers, healthcare providers, and government health departments to enhance traditional surveillance for detecting, monitoring, reporting, and responding to influenza, dengue, and COVID-19.

**Supplementary Information:**

The online version contains supplementary material available at 10.1007/s44197-024-00272-y.

## Introduction

As we move forward managing COVID-19 alongside other communicable diseases, following the World Health Organization declaring the end of the global health emergency in May 2023 [1]. Widespread public health measures have now ended [2] as the immediate threat of COVID-19 has faded following a successful global vaccination effort and the occurrence of less severe variants circulating [3] although the aftermath of the pandemic including loss of life, and economic and societal upheaval endures and will do so for many years to come. COVID-19 is now considered endemic in humans alongside seasonal influenza and respiratory diseases such as respiratory syncytial virus (RSV), human metapneumovirus (HMPV) and others, while the risk of more virulent or vaccine-avoidant variants of SARS-CoV-2 or other viruses causing severe outbreaks in the future remains [4]. 

In recent years, with the increasing effects of climate change and other environmental and land use changes, the threat of emerging infectious diseases (EIDs) from spillover events has grown amidst warnings from experts [5]. Over the last decade [6], recent epidemics/pandemics caused by zoonotic spillover events have occurred [7], Avian influenza (H7N9) (2013-17), Swine flu (H1N1) (2009-10) alongside increasing occurrence and range of mosquito-borne diseases including Japanese Encephalitis virus (Australia 2022), Zika virus (2015–2016), and Dengue fever (continuous and ongoing in many countries) [8]. The emergence of the novel coronavirus in December 2019 leading to the COVID-19 pandemic has led to a renewed interest in disease surveillance and outbreak tracking with the need for integrative digital early warning and surveillance systems more pronounced than ever [9]. 

The COVID-19 pandemic caused significant disruption to global health, with widespread impacts on existing public health measures, vaccination and prevention programs and community surveillance programs for many diseases. In particular, pandemic response measures for the control of COVID-19 including non-pharmaceutical interventions (i.e., hygiene measures, masking, social distancing) [10] and travel restrictions (border closures and movement restrictions) contributed to decreased influenza circulation and other respiratory disease activity during this time [11–13]. These actions have resulted in the global disappearance of Influenza B/Yamagata strain [14], and altered patterns of dengue outbreaks in non-endemic countries [15], even as overall annual incidence and distribution have increased [16]. 

While research interest in digital surveillance has remained high over the previous decade, particularly for influenza outbreak detection [17], the COVID-19 pandemic was an unprecedented opportunity to employ methods in real-time to detect outbreaks, forecast epidemic growth and tailor effective and locally relevant public health messaging [18, 19]. Internet-based or digital surveillance systems [20], use online data sources to detect digital signals for potential indicators or early signs of infectious disease outbreaks based on online information seeking and trends in user behaviours from a range of social media and search engine sources. Predictive modelling and forecasting use digital signals to estimate the risk of an outbreak, rate of transmission or forecast the spread of disease. By analysing large volumes of online data in real-time, predictive models can be used to identify high-risk clusters, trends and early warning signs often preceding traditional surveillance methods for disease detection (i.e., laboratory-confirmed testing or diagnosis in a healthcare setting) and can provide early warning of outbreaks prior to these health system alerts, and are complementary to event-based electronic surveillance systems such as GPHIN and ProMED [21]. Over the last decade, there have been many changes in the online ecosystem, with emerging social media platforms, changing user behaviours, and the emergence of AI chatbots integrated into search engines and social media, blurring the lines between sources of information. With the emergence of and widespread implementation of Artificial Intelligence (AI), as described in recent reviews including Brownstein et al. [22], and Macintyre et al. [23], the potential applications in the digital space continue to grow, alongside increased computing capability and signal detection to improve the speed and capacity of existing EWS and surveillance, enabling earlier detection to manage serious epidemics and pandemics in the future [24]. 

### Objectives

In this review, we evaluated studies from the past decade (2013–2024) to capture changing trends in digital surveillance for influenza, COVID-19 and dengue, as representative of broader respiratory and vector-borne diseases with high levels of surveillance. We describe the changes over time in digital surveillance, and forecasting for selected infectious diseases since our previous review [20], the advantages and limitations of using digital surveillance data, and advances in AI and digital technology. Due to the increasing range of social media and search engine data sources and increased integration of multiple data sources the included studies have been grouped by disease of interest rather than data source, these are summarized in Table S2. Finally, we make recommendations for future research into digital surveillance for useful early warning systems (EWS).

## Methods

Using a systematic review approach, we searched PubMed and Scopus databases for peer-reviewed original research publications between July 1, 2013, and March 31, 2024, according to PRISMA 2020 guidelines (See Supplementary Table S1) [25]. Additional relevant publications were identified from references.

### Search Strategy and Selection Criteria

We performed searches with the following terms: “dengue” OR COVID-19 OR “influenza” AND “early warning”, “Google”, “Google Trends”, “internet”, “search engine”, “social media”, “Twitter”, “Facebook”, OR “digital disease detection”, “infodemiology”, “infoveillance”, “real-time disease surveillance”, and “syndromic surveillance”. To be eligible for inclusion, studies needed to be peer-reviewed and describe the use of internet-based data for surveillance, predictive modelling, forecasting or early warning for influenza, COVID-19, or dengue. Studies were excluded if they were not original research, did not include digital data sources (social media or search trends), or did not discuss influenza, COVID-19, or dengue. Mathematical and computational modelling studies using simulated data were excluded. Due to the evolving nature of AI in its current form and limited access to user data, no studies were published that the authors are aware of at this time. The data for full-text screened articles were extracted and summarized See Supplementary Table S2. Due to varying study designs, methodologies, models, statistical analysis and potential confounders, no meta-analysis was performed.

## Results

**Fig. 1:**
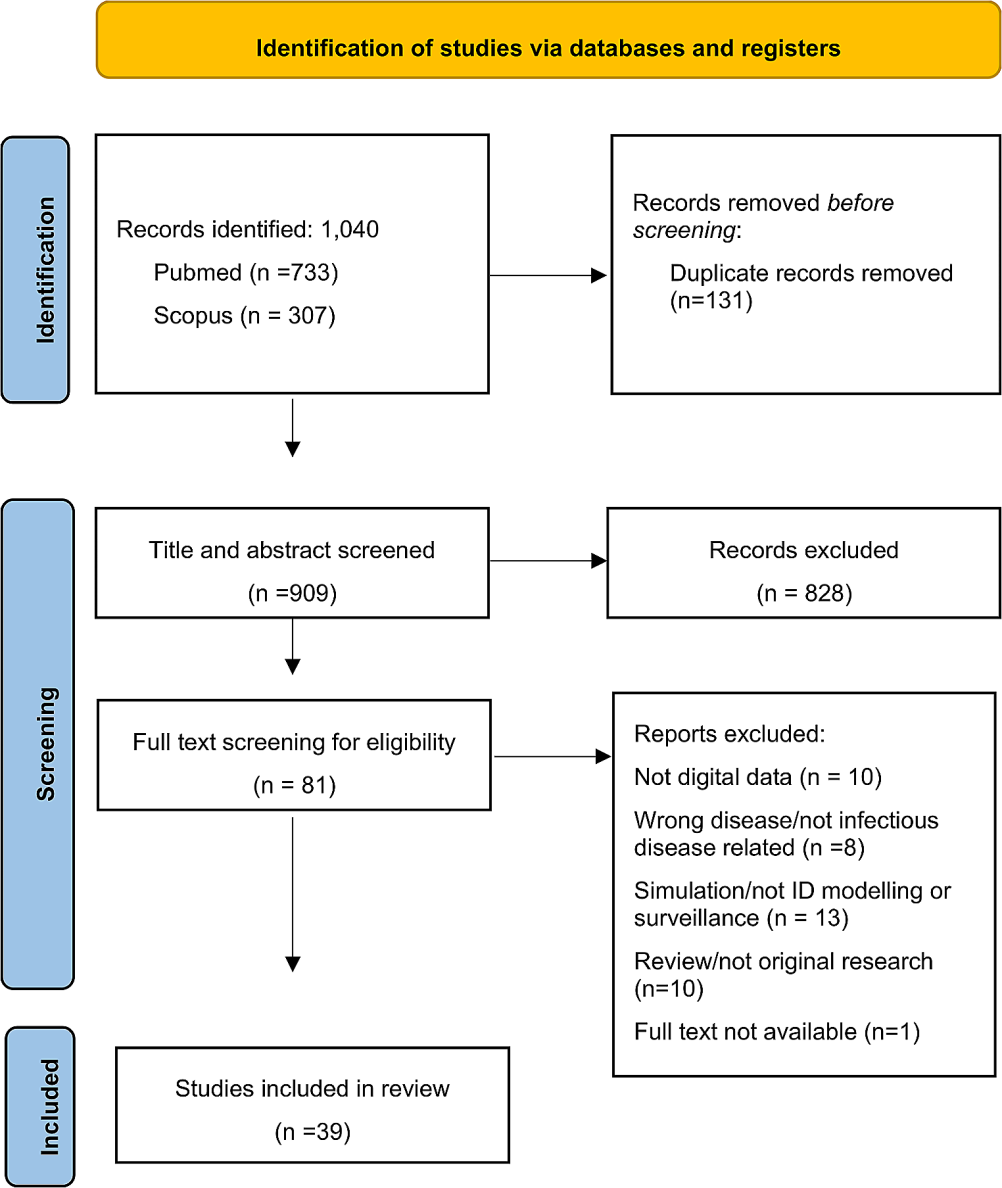
Flowchart with article selection

A total of 1040 studies were identified through the literature search and reference checking, and 131 duplicate records were excluded (Fig. 1). The remaining 909 papers were assessed for eligibility and screened by checking the title and abstract for relevance. Subsequently, 828 papers were excluded, leaving 81 papers for full-text review where references were checked for additional relevant papers, 43 full-text papers were excluded.

**Fig. 2:**
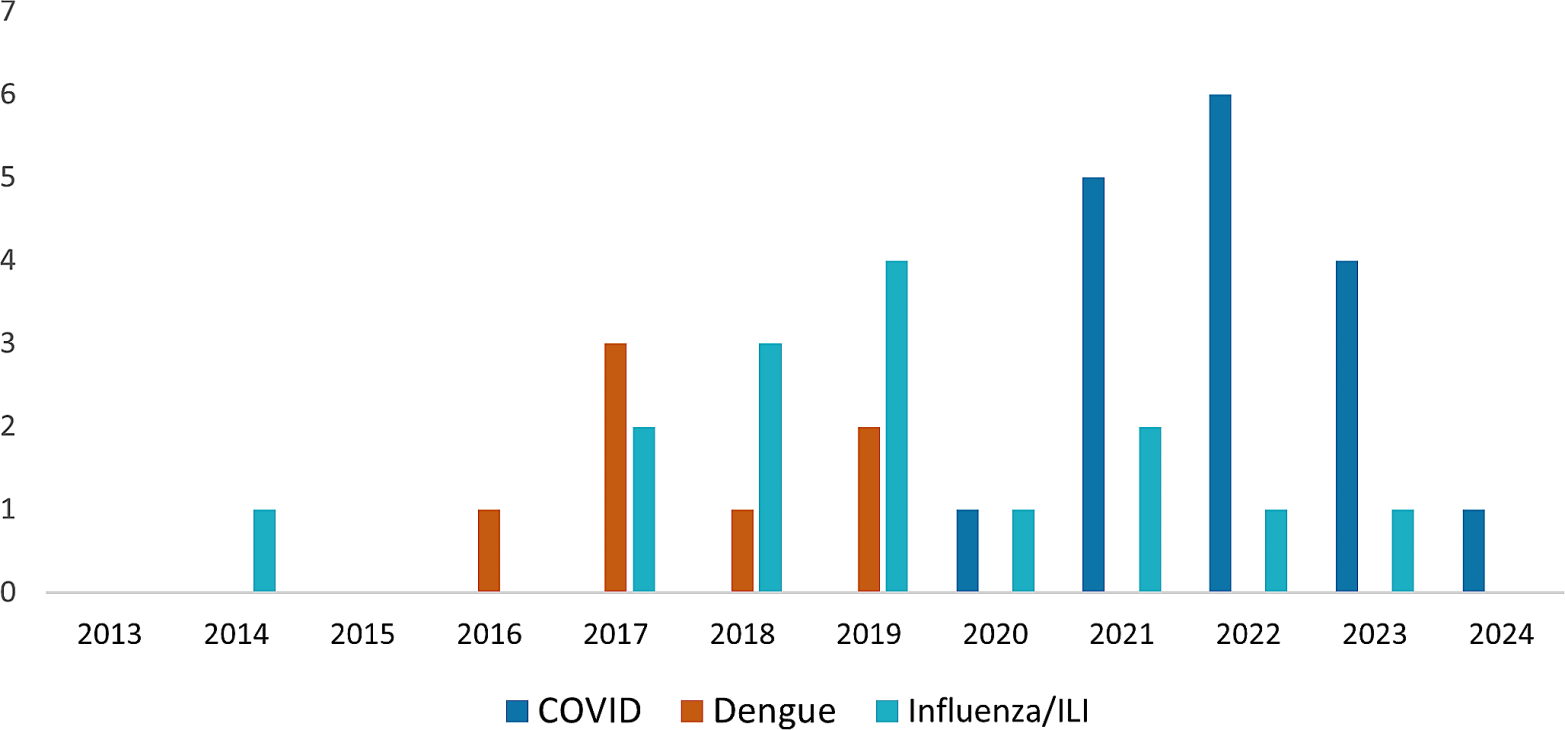
Number of selected articles by year of publication and disease of interest from PubMed and Web of Science using digital big data for early warning, surveillance and predictive modelling (Data up to March 2024)

Of the 39 selected studies, 17 focused on COVID-19, 15 studies described influenza or influenza-like illnesses (ILI), and 7 studies described dengue fever (Fig. 2). Digital data sources included Google Search Trends and Community Mobility, Apple Mobility, Baidu Search (the main search engine used in China), Bing Search, Wikipedia, and social media including X (formerly known as Twitter), Weibo and WeChat in China (equivalent to Twitter/X and WhatsApp). Models and methodologies varied ranging from simple correlation (Pearson’s or Spearman’s Rank Correlation) and Time Series Cross Correlation to spatial and temporal models including Poisson linear regression, generalized linear models, predictive models including SARIMA and ARIMA, LSTM, Prophet and SVR through to machine learning, NLP and neural networks. All these models have varying strengths and limitations, certain models may be more appropriate in specific situations, particularly when considering computational complexity for low resource settings, further discussion on modelling for EWS can be found in Haque et al. [26]. 

### Disease Specific Internet-Based Surveillance

#### Influenza and ILI Surveillance

This review identified research using search queries for surveillance and early warning for influenza/ILI from Mexico [27], the United States [28, 29], Australia [30], Hong Kong [31], South Africa [32], and a multi-country study [33]. These studies reported Google search results with climate and weather variables included in models enhanced predictive accuracy and emerging outbreaks and seasonal variations for ILIs and respiratory diseases were detected weeks earlier compared with conventional surveillance [34]. Studies from China utilised Baidu search, Guo et al. proposed a surveillance framework based on significant keywords [35], Chen et al. [36] used seasonal SARIMA models with Baidu search (*β* = 0.008, *p* < 0.001) and Weibo (*β* = 0.002, *p* = 0.036) for search terms “H7N9”, “avian influenza”, and “live poultry” to explore the association with weekly laboratory-confirmed H7N9 cases. Yang et al. [37] developed deep learning prediction models for ILI, reporting that integrating both climate factors and search trends enhanced model accuracy. Additionally, Google and Baidu search queries were used to forecast seasonal influenza outbreaks cross-hemisphere for the United States, United Kingdom, and China using Australian Influenza surveillance data. The resulting SARIMA models demonstrated high correlation coefficients (China = 0.96, the US = 0.97, the UK = 0.96, *p* < 0.01) and low Maximum Absolute Percent Error (MAPE) values (China = 16.76, the US = 96.97, the UK = 125.42), significantly improving predictive accuracy over case-only models [38]. 

Beyond search queries, text mining of symptom keywords on social media enabled near real-time syndromic surveillance for flu/unwell in Australia [39], identifying changes in frequency counts compared to public health notifications. Natural Language Processing was used to detect avian influenza notifications, by processing Tweets, 75% of official outbreak notifications (i.e. farm records, outbreaks and individual cases) were identified from the sample dataset, and a third of these detections were identified earlier than official notifications [40]. Using Twitter/X geolocation data, Nagar et al [41]. used tweet vector maps to identify clusters of ILI in New York providing insight into spatiotemporal patterns of ILI. Another study [42] found there was a strong temporal association with flu-related Tweet activity for healthcare seeking behaviour preceding official reports of influenza cases by up to one month, and identified hotspots related to public spaces.

#### Dengue Fever (DF) Surveillance

While there were fewer studies on internet-based surveillance for DF compared with Influenza and COVID-19, the magnitude of dengue outbreaks significantly increased in 2023, compared with the previous periods from 2018 to 2022. This may potentially be attributable to public health interventions during the pandemic response impacting transmission and testing availability and research focus on COVID-19 over this time [43]. These studies covered China, Brazil, the Philippines, and Indonesia. In China, researchers used Baidu Search trends for dengue to determine thresholds to detect outbreaks. Weekly search indexes showed a positive correlation with incidence rates, and a lagged moving average of 1–3 weeks greater than 99.3, indicated there was an 89.28% chance of an outbreak in Guangzhou. In Zhongshan, weekly BSI at 1–5 weeks was over 68.1 with the chance of an outbreak increased by 100% [44]. In Guangdong province, Guo and co-authors developed a forecasting model using Baidu search queries and weather factors, with support vector regression (SVR) model consistently demonstrating lower prediction error rates [45]. 

Liu et al. reported increased predictive accuracy of models using weather, Baidu search and demographics in Guangzhou city [46]. Li et al [47]. found that dengue-related searches at a lag of one week were positively correlated with DF occurrence. The model including search indexes had greater predictive capability (ICC:0.94, RMSE:59.86) compared to the model without search data (ICC:0.72, RMSE:203.29). Ho et al. explored temporal and spatial patterns of dengue incidence in Manila, Philippines, using Google Dengue Trends (GDT). Weekly values of DF incidence were moderately associated (*r* = 0.405) with weekly GDT values, while spatial analysis was not significant (*r* = 0.223, *p* = 0.283) [48]. In Indonesia, Husnayain et al. reported a significant correlation with Google search terms for dengue symptom, dengue and dbd (dengue abbreviation) showing the highest correlation one week preceding; *r* = 0.937, 0.931 and 0.921 respectively (*p* ≤ 0.05) [49]. 

Finally, Marques-Toledo and co-authors explored multiple digital data sources for estimating and forecasting DF at the city level in Brazil. They accessed Google Search, X and Wikipedia to explore real-time health-seeking behaviour for DF. The authors reported a positive linear association with Tweets (*r* = 0.87, *p* < 0.001), Google Trends (*r* = 0.92, *p* < 0.001), and Wikipedia (*r* = 0.71, *p* < 0.01). Tweets selected by city were used to develop nowcasting and forecasting models, demonstrating temporally association with DF up to 8 weeks in advance, with the strongest association at lag week 1 [50]. 

#### COVID-19 Pandemic

From the initial detection of COVID-19 in December 2019 to the present, the novel pandemic situation received significant attention and resulted in extensive epidemiological research. Early research concentrated on the initial outbreak and the first wave in China, using search queries (Baidu) and social media/microblogs (Weibo, WeChat and Douyin/TikTok) to detect early signals of the emerging pandemic as people searched for the latest news and updates on escalating outbreaks [51]. 

Studies using Baidu search data found search terms associated with COVID-19 valuable for early outbreak warning. Tu et al. reported an average “search to confirmed interval” of 19.8 days, with optimal time lags for search queries at 0–4 days [52]. Li et al. reported significant lags at 4–7 days preceding conventional surveillance identification, with early warning signs up to 20 days earlier than lockdown policy implementation [53]. Integrating multiple data sources enhanced predictive accuracy and early warning capabilities. Gong et al. compared search interest and microblogs for daily new cases, new deaths and outbreak severity, revealing advanced trends between lags of 3–16 days for both Baidu and Weibo [54]. Baidu and Weibo showed a significant positive correlation with cases and deaths, with Baidu search having a stronger correlation.

Weibo keyword trends for symptoms and diagnosis were useful for detecting early signals of COVID-19. Guo et al. used Weibo data to improve the predictive accuracy of early epidemic models and Shen et al. used social media data to accurately identify early digital disease signals [55, 56]. Li et al. combined diverse internet data, including online news articles, microblogs and search trends to improve model forecasting accuracy providing early warning signals for outbreaks 2 to 6 days in advance [57]. Gao et al. utilized search queries and video-based social media Douyin keyword trends to predict asymptomatic or undetected transmission [58], capturing data from younger subpopulations.

As COVID-19 spread globally, international travel restrictions and public health measures were implemented following the WHO pandemic declaration [59]. The number of studies using digital signals grew quickly, with an English study using Bing search to detect early warning of COVID-19 [60], and X data to explore symptom keywords for early detection of COVID-19 in Europe and globally [61, 62]. During this time, there was an increasing use of multisource digital data, combining search trends and social media data. Twitter/X data proved useful for providing early warning of outbreaks in studies from the United States and Canada [63–65]. Symptom-based keyword searches for both Google search and X were temporally correlated, with Google searches for “cough,” “runny nose,” and “anosmia” correlated with COVID-19 incidence and peaked 9, 11, and 3 days earlier than the incidence peak, respectively. This improved the predictive accuracy of LSTM forecasting models (MSE = 124.78, R^2^ = 0.88) [66]. In California, Habibdoust *et al* used search volumes for “Fever,” “COVID Testing,” “Signs of COVID,” “COVID Treatment,” and ”Shortness of Breath” to predict daily incidence comparing GMDH-type neural network and LSTM models over three time periods [67], where models with queries improved predictive accuracy by as much as 22.6%, 21%, and 37.3% in NRMSE across the different study periods.

In our study from Victoria, Australia, we used Google Mobility as a proxy for population movement and non-pharmaceutical interventions on COVID-19 transmission, integrating search trends and weather factors to forecast epidemic growth [68]. The multivariable weather and mobility model demonstrated the highest predictive accuracy (R^2^ = 0.948, RMSE = 137.57, MAPE = 21.26) compared to cases only (R2 = 0.942, RMSE = 141.59, MAPE 23.19). Finally, Kogan et al. developed an integrated EWS for detecting ILI globally, monitoring COVID-19 activity using multiple digital sources including Google search trends, Apple Mobility, Twitter/X API with ILINet (CDC sentinel system) and UpToDate physician search trends and smart thermometer data and found digital proxies for COVID-19 preceded detection through normal clinical surveillance [69]. 

## Discussion

### Brief Overview of EWS

Within the broader scheme of global outbreak detection and pandemic preparedness, timely disease detection and notifications are limited by aging infrastructure and reduced public health funding leading to downgrading or discontinuation of existing early warning surveillance systems. In the lead-up to the detection of atypical pneumonia and official reports to the WHO in December 2019–January 2020, the US dissolved the National Security Council (NSC) Directorate for Global Health Security and Biodefense in May 2018, responsible for monitoring global health risk and coordinating government response [70]. As the oldest digital surveillance system, ProMED-mail has provided reliable event-based surveillance for over 25 years [71], and successfully detected the first alert of COVID-19 in December 2019. Despite playing an important role in global public health surveillance, as with many pandemic preparedness tools, ProMED-mail suffers from funding shortages reducing the availability and capacity for detecting future outbreaks. Another well-known digital surveillance system, the Global Public Health Intelligence Network (GPHIN) [72], an event-based EWS developed by the Canadian Government to collect global and multilingual media reports to create alerts in the wake of SARS. Having successfully detected early signs of outbreaks including the Middle East Respiratory Syndrome (MERS), influenza pandemics, and Zika virus, leading up to the COVID-19 detection, GPHIN alerts were increasingly limited by outdated system capabilities and downgraded reporting and response.

### Emerging Trends in Digital Surveillance from the COVID-19 Pandemic

In the last decade, research consistently supports the use of internet-based data for infectious disease surveillance, with continued research using these methods, though more recent studies often utilised multi-source digital data i.e., both search queries and social media and an expanded range of sources. This approach offers advantages in capturing information from difficult-to-reach sources beyond the medical system, including individuals seeking testing, accessing health resources, or being hospitalized. Digital surveillance excels at detecting early signals from recently exposed individuals, those with milder disease states, and younger demographics with limited access to regular healthcare or a lower inclination to seek care. Digital data provides near real-time availability, facilitates discussions about symptoms or keywords with geotagging functionality, and links online behaviours to unique user accounts and online networks, potentially reflecting real-world connections like family, friends, and co-workers.

**Fig. 3:**
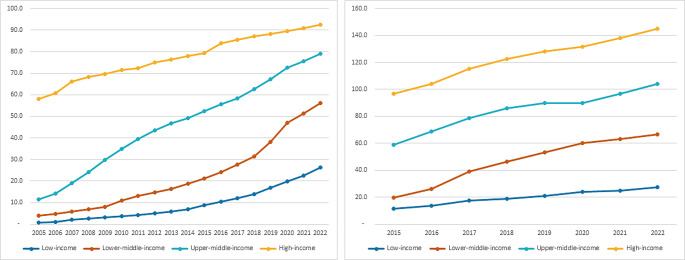
Individuals using the Internet per 100 population (Left) Active mobile-broadband subscriptions per 100 population (Right) (Data not available before 2015) by global development status. (Data Source: https://datahub.itu.int/). (Source: ITU)

Over the last decade, there have been many technological advances affecting internet usage and digital health care seeking. Levels of internet access in the home have increased alongside significant growth in smartphone usage since 2013, numbers of which are higher in advanced economies compared to regions with higher rates of poverty (see Fig. 3). Along with the proliferation of smart devices or the Internet of Things (IoT), GPS-enabled and WIFI-connected wearables and mobile devices are widespread and able to collect biometric, audio and location data in real-time potentially able to diagnose IDs in pre-symptomatic infected individuals through biosignals or distinctive COVID-19 or pneumonia coughing [73].

#### Changing Digital Landscape and Data Availability

Along with the continuous 24-hour news cycle and inundation of health information over a range of platforms; the way users consume and interact with media for healthcare-seeking and news has shifted over the last decade. Digital usage trends since the early 2020 pandemic lockdowns contributed to changing online behaviours and increased digital communication. While social media is a useful medium for targeted public health messaging where users are increasingly accessing news and health advice, information from official sources appears alongside content containing misinformation and disinformation [74]. With the emergence of AI, unlimited access by LLM for training data has led to restrictions on data from many of the previously freely available data sources. Over time the scope and availability of aggregate data sets have changed, these changes will significantly impact digital research, affecting applications in short- and long-term trend analysis for pandemic detection, disaster responses during social unrest, and extreme weather events [75]. 

While the potential applications for AI are evolving and changing rapidly, recent changes to integrate AI chatbots into search engines and social media i.e., Microsoft Bing and Google search engines powered by AI chatbots and Meta AI and X-AI on social media platforms are shaping online user behaviour [76], meaning search results and social media algorithms are trained on user behaviour aiming to provide faster, more accurate and relevant answers based on user profiles and behaviour across the online ecosystem [77]. Using AI trained on user-provided information across the digital landscape to answer user queries rather than directing traffic to websites will contribute to changes in the consumption of social media as a news source and information seeking, where users are increasingly searching and accessing news and information from social media sources, i.e., health and government information releases and media coverage in real time, meaning the distinction between digital signals from search engines and posting about symptoms on social media are increasingly blurring.

#### Integrating AI Technology and Advances in Digital Data

Emerging technological advances are accelerating hand in hand with changing usage trends, with potential uses in future research and applications in healthcare quickly evolving [78]. The growth of AI and machine learning algorithms and increased computing capability allow for processing more complex and larger data sets, faster data mining, and improved capacity for predictive models. With increased capability to deal with high volumes of text-based communications including surveillance reports and news coverage for identifying and classifying infectious disease signals and detecting epidemics [79]. 

Evaluating the usefulness of digital data sources is essential where some sources may contain a greater amount of noise and, positive signals can overwhelm the capacity of a system to recognise and respond to events in real-time. Ensuring data fidelity where data is captured accurately, with precision and timeliness, is essential [80]. This can be achieved through a range of methods including real-time monitoring and constant sampling to improve the detection of data signals and show complex patterns and changes over time can improve signal detection accuracy and are less prone to noise and artifacts in the data [81]. Methodological approaches including multisource data and improved machine learning and neural networks can improve data fidelity, alongside real-world reporting and test results to validate and fine-tune models for best results, and is an important consideration in future research.

How users are interacting with AI chatbots, as an alternative to internet searches for self-diagnosis, may have the potential to transform healthcare, by capturing the healthcare-seeking actions of internet users to detect early warning signals [82]. Applications include the use of chatbots trained on medical research for answering consumer health questions as natural language alternatives to conventional keyword-based search methods for healthcare seeking [83, 84], as mHealth (mobile health) applications with virtual educator interface for providing health information [85], diagnostic purposes and assessing illness severity.

### Developing Integrative Multisource Early Warning Systems

Developing integrated multisource EWS holds significant potential for improving the early detection of disease signals and identifying emerging outbreaks, particularly for climate and weather-sensitive vector-borne or respiratory diseases. This is particularly important in regions experiencing increasing climate stress, at greater risk of spillover events, and where the population may experience high levels of poverty or limited access to healthcare infrastructure. Our previous research has emphasised the usefulness of incorporating socio-environmental factors including weather with internet-based data across a range of diseases [86, 87]. Additionally, utilizing Geographical Information Systems (GIS) at high spatial resolution for climate-sensitive or vector-borne diseases [88], has the potential to enhance predictive modelling and early warning for outbreaks [89]. 

**Fig. 4:**
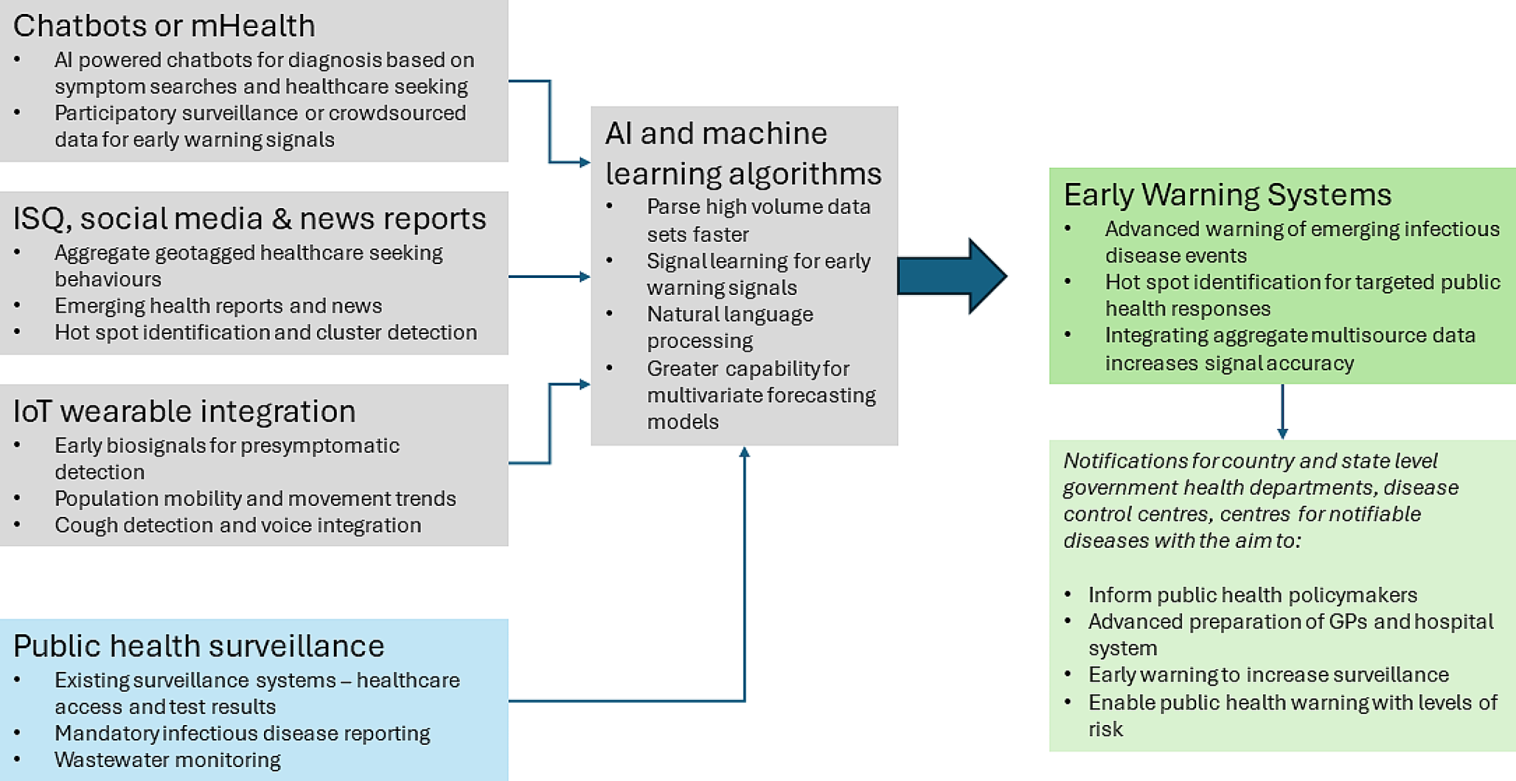
Integrating multisource internet-based data with existing surveillance methods using AI and machine learning for improving early warning of infectious diseases

Using existing surveillance and wastewater monitoring, mHealth applications with AI chatbots, internet-based data and socio-environmental factors can be useful for the timely detection and notification of emerging outbreaks, identifying spatiotemporal variations in outbreaks or hot spots or high-risk clusters. An EWS would facilitate timely public health notifications to the public and assist health staff and policymakers with broader applications in detecting mass gatherings and emergencies (See Fig. 4). Some related examples of this include EpiWatch [79], an AI-based system providing early warnings of epidemics on a global scale based on open data, and HealthMap (https://www.healthmap.org), using natural language processing and Bayesian machine-learning classification trained to identify relevant information from digital data and PROMED-mail [71] alerts to identify infectious disease outbreaks.

### Advantages and Limitations of Digital Surveillance

Digital data for infectious disease surveillance remains an important area of research. This approach offers advantages in capturing information from hard-to-reach sources outside the medical system i.e., people seeking testing, accessing health resources or hospitalised. Digital surveillance is useful for detecting signals from recently exposed individuals, those experiencing milder disease states, and younger demographics with limited access to regular healthcare or less likely to seek care. Digital data has near real-time availability, enables discussion of symptoms or keywords with geotagging functionality, and connects online behaviours to unique user accounts and networks, potentially representing real-world connections with family, friends, and co-workers [90]. However, the digital divide among social media users based on age may lead to under-detection for disease surveillance purposes.

While internet-based surveillance holds significant potential for EWS, there are limitations to consider. Although internet access has improved, coverage remains limited in LMIC, reducing early signal detections for infectious disease surveillance, particularly in rural or remote areas [91]. Post-COVID changes in laboratory diagnostics and respiratory disease surveillance may impact the future utility of these methods. Spatial resolution is limited [92], with limited availability of city-level data. Data sets are often discontinued or monetised, measurement indexes may have accuracy issues over time, potentially impacting research reproducibility across various geospatial contexts [93]. 

As Internet usage has increased, so has the availability of digital data. However, interpreting signals and predictive values with appropriate sensitivity and specificity, and distinguishing true signals from false positives or noise, remains a significant challenge. Moreover, as internet usage has increased and data linkage raises ethical concerns about accessing and using personal health information [94, 95] with potential risks for user safety and privacy, care must be taken to ensure patient and individual privacy and personal data are protected. Finally, the current generation of AI chatbots have a tendency to ‘hallucinate,’ [96] providing incorrect or nonsensical responses which is particularly dangerous in healthcare scenarios.

### Future Research

Future research must focus on a better understanding of digital health signals and digital surveillance, the growing use of AI, and how these are changing the online environment in terms of healthcare information-seeking and collation of web-available data. Finally, an informative and useful EWS based on an integrated framework including conventional surveillance data, crowdsourced surveillance, and a wide range of internet-based surveillance sources to build the capacity of existing surveillance for the future requires global cooperation for information and resource sharing.

In summary, for improving infectious disease early warning surveillance we recommend:

1. Development of novel dynamic GIS-based spatiotemporal models to link infectious diseases with internet-based data and social-environmental data.
2. Integration of internet-based models with social-environmental data to produce infectious disease surveillance systems able to better identify vulnerable/susceptible communities over space and time.
3. Developing innovative mHealth applications with a chatbot and virtual educator interfaces.
4. Expand the knowledge of big data utility in infectious disease early warning systems and development of functional infectious disease early warning systems, minimising noise and ensuring real signals are identified early and accurately without overwhelming existing system capacity.

## Conclusion

With increasing internet access globally, the world is more connected digitally than ever before as the availability and range of digital data sources available for disease surveillance have grown. Our increased interconnectedness and globalisation as COVID-19 swept through the world underlined the importance of consistent global surveillance, for the detection and reporting of such events. The next 10 years will be a challenging period, and it remains to be seen if we can indeed achieve the holy grail of being able to forecast existing or newly emerging diseases with accuracy and early enough to allow measures to be enacted that will mitigate or halt these outbreaks.

## Electronic supplementary material

Below is the link to the electronic supplementary material.

Supplementary material 1

## Data Availability

No datasets were generated or analysed during the current study.
