# Supplementary material for: Internet-based Surveillance Systems and Infectious Diseases Prediction: An Updated Review of the Last 10 Years and Lessons from the COVID-19 Pandemic
Source: J Epidemiol Glob Health. 2024 Aug 14;14(3):645–57. doi: 10.1007/s44197-024-00272-y (PMC11442909; doi:10.1007/s44197-024-00272-y)
Supplement: Supplementary file 1 — Supplementary material 1 [file 44197_2024_272_MOESM1_ESM.docx]

# Supplementary Material

Table S1: PRISMA checklist

| **Section and Topic** | **Item #** | **Checklist item** | **Location where item is reported** |
| --- | --- | --- | --- |
| **TITLE** | | |  |
| Title | 1 | Identify the report as a systematic review. | 1-2 |
| **ABSTRACT** | | |  |
| Abstract | 2 | See the PRISMA 2020 for Abstracts checklist. | 30-41 |
| **INTRODUCTION** | | |  |
| Rationale | 3 | Describe the rationale for the review in the context of existing knowledge. | 67-89 |
| Objectives | 4 | Provide an explicit statement of the objective(s) or question(s) the review addresses. | 90-98 |
| **METHODS** | | |  |
| Eligibility criteria | 5 | Specify the inclusion and exclusion criteria for the review and how studies were grouped for the syntheses. | 102-112 |
| Information sources | 6 | Specify all databases, registers, websites, organisations, reference lists and other sources searched or consulted to identify studies. Specify the date when each source was last searched or consulted. | 102-112 |
| Search strategy | 7 | Present the full search strategies for all databases, registers and websites, including any filters and limits used. | 102-112 |
| Selection process | 8 | Specify the methods used to decide whether a study met the inclusion criteria of the review, including how many reviewers screened each record and each report retrieved, whether they worked independently, and if applicable, details of automation tools used in the process. | 102-112 |
| Data collection process | 9 | Specify the methods used to collect data from reports, including how many reviewers collected data from each report, whether they worked independently, any processes for obtaining or confirming data from study investigators, and if applicable, details of automation tools used in the process. | 102-112 |
| Data items | 10a | List and define all outcomes for which data were sought. Specify whether all results that were compatible with each outcome domain in each study were sought (e.g. for all measures, time points, analyses), and if not, the methods used to decide which results to collect. | 102-112 |
|  | 10b | List and define all other variables for which data were sought (e.g. participant and intervention characteristics, funding sources). Describe any assumptions made about any missing or unclear information. | 102-112 |
| Study risk of bias assessment | 11 | Specify the methods used to assess risk of bias in the included studies, including details of the tool(s) used, how many reviewers assessed each study and whether they worked independently, and if applicable, details of automation tools used in the process. |  |
| Effect measures | 12 | Specify for each outcome the effect measure(s) (e.g. risk ratio, mean difference) used in the synthesis or presentation of results. | N/A |
| Synthesis methods | 13a | Describe the processes used to decide which studies were eligible for each synthesis (e.g. tabulating the study intervention characteristics and comparing against the planned groups for each synthesis (item #5)). | N/A |
|  | 13b | Describe any methods required to prepare the data for presentation or synthesis, such as handling of missing summary statistics, or data conversions. | N/A |
|  | 13c | Describe any methods used to tabulate or visually display results of individual studies and syntheses. | 121 |
|  | 13d | Describe any methods used to synthesize results and provide a rationale for the choice(s). If meta-analysis was performed, describe the model(s), method(s) to identify the presence and extent of statistical heterogeneity, and software package(s) used. | N/A |
|  | 13e | Describe any methods used to explore possible causes of heterogeneity among study results (e.g. subgroup analysis, meta-regression). | N/A |
|  | 13f | Describe any sensitivity analyses conducted to assess robustness of the synthesized results. | N/A |
| Reporting bias assessment | 14 | Describe any methods used to assess risk of bias due to missing results in a synthesis (arising from reporting biases). | N/A |
| Certainty assessment | 15 | Describe any methods used to assess certainty (or confidence) in the body of evidence for an outcome. | N/A |
| **RESULTS** | | |  |
| Study selection | 16a | Describe the results of the search and selection process, from the number of records identified in the search to the number of studies included in the review, ideally using a flow diagram. | Figure 1 |
|  | 16b | Cite studies that might appear to meet the inclusion criteria, but which were excluded, and explain why they were excluded. | N/A |
| Study characteristics | 17 | Cite each included study and present its characteristics. | Appendix (Supp. Table 2) |
| Risk of bias in studies | 18 | Present assessments of risk of bias for each included study. | N/A |
| Results of individual studies | 19 | For all outcomes, present, for each study: (a) summary statistics for each group (where appropriate) and (b) an effect estimate and its precision (e.g. confidence/credible interval), ideally using structured tables or plots. | N/A |
| Results of syntheses | 20a | For each synthesis, briefly summarise the characteristics and risk of bias among contributing studies. | N/A |
|  | 20b | Present results of all statistical syntheses conducted. If meta-analysis was done, present for each the summary estimate and its precision (e.g. confidence/credible interval) and measures of statistical heterogeneity. If comparing groups, describe the direction of the effect. | N/A |
|  | 20c | Present results of all investigations of possible causes of heterogeneity among study results. | N/A |
|  | 20d | Present results of all sensitivity analyses conducted to assess the robustness of the synthesized results. | N/A |
| Reporting biases | 21 | Present assessments of risk of bias due to missing results (arising from reporting biases) for each synthesis assessed. | N/A |
| Certainty of evidence | 22 | Present assessments of certainty (or confidence) in the body of evidence for each outcome assessed. | N/A |
| **DISCUSSION** | | |  |
| Discussion | 23a | Provide a general interpretation of the results in the context of other evidence. | 264-364 |
|  | 23b | Discuss any limitations of the evidence included in the review. | 365-388 |
|  | 23c | Discuss any limitations of the review processes used. | 365-388 |
|  | 23d | Discuss implications of the results for practice, policy, and future research. | 390-400 |
| **OTHER INFORMATION** | | |  |
| Registration and protocol | 24a | Provide registration information for the review, including register name and registration number, or state that the review was not registered. | N/A |
|  | 24b | Indicate where the review protocol can be accessed, or state that a protocol was not prepared. | N/A |
|  | 24c | Describe and explain any amendments to information provided at registration or in the protocol. | N/A |
| Support | 25 | Describe sources of financial or non-financial support for the review, and the role of the funders or sponsors in the review. | 421-422 |
| Competing interests | 26 | Declare any competing interests of review authors. | 419-420 |
| Availability of data, code and other materials | 27 | Report which of the following are publicly available and where they can be found: template data collection forms; data extracted from included studies; data used for all analyses; analytic code; any other materials used in the review. | N/A |

Table S2: Data extraction table for included studies.

| **Authors** | **Data Source (resolution; range)** | **Disease surveillance** | **Region** | **Methodology** | **Results** | **Study Limitations** |
| --- | --- | --- | --- | --- | --- | --- |
| Chen *et al* (2019)[36] | Baidu Search Index (BSI) & Weibo Index (WI) (weekly; 2013 - 2017) | Avian Influenza (H7N9) | China | The authors correlated H7N9 influenza related search query data ("H7N9", "avian influenza" and "live poultry”) and social media data and mapped distributions of search interest at the provincial level with seasonal decomposition to describe seasonal trends. Poisson linear regression model was performed to assess spatial dispersion between cases numbers and BSI. Time series cross correlation was used to determine lags for SARIMA predictive modelling using BSI and Wito predict cases, CART was used to determine threshold effects. | Weekly H7N9 case occurrence was positively correlated with weekly BSI with a lag of −2 to +3 weeks for the search term “H7N9”, a lag of −3 to +3 weeks for the search term “Avian influenza”, and a lag of −4 to +7 weeks for the search term “Live poultry” and positively correlated with weekly WPI with a lag of −2 to +1 weeks for the search term “H7N9”, with a lag of −3 to 0 weeks for “Avian influenza”, and a lag of −3 to 0 weeks for “Live poultry”. | Limited spatial resolution, data only available at province level. |
| Didi *et al*  (2022)[62] | Twitter (Daily; March 2020 - Feb 2022) | COVID-19 | Global | The authors correlated COVID-19 Twitter data with cases using Spearman's rank and Pearson correlation, to compare machine learning models for feature extraction and compare univariate and multivariate predictive models to forecast COVID-19 case curves using LSTM, Prophet and SVR and in relation to Twitter data. | Incorporating multivariate data, including social media data improved the forecasting accuracy where *r* value is equal to 0.98 for the LSTM multivariate, 0.97 for the Prophet multivariate and 0.952 for multivariate SVR. Machine learning models displayed differing levels of accuracy, precision and recall for feature extraction. | Tweet language limited to English, spatial resolution at country level only |
| Fan *et al* (2022)[31] | Google search | Swine flu (H1N1) | Hong Kong | The correlation between ED patient arrivals and Google search data was explored and applied to 8 forecasting models to predict ED patient arrivals, including Extreme Learning Machine (ELM), generalized linear model (GLM), autoregressive integrated moving average model (ARIMA), ARIMA with explanatory variables (ARIMAX), support vector machine (SVM), artificial neural network (ANN), random forest (RF), and long short-term memory (LSTM) | ED patient arrivals were significantly correlated search index (r=0.46, P=.002). Including search queries improved prediction accuracy over patient arrivals, and arrivals with other factors. Machine learning (ELM) model had higher accuracy, Including internet search index results in more stable models. | Limited to single hospital ED, local population specific search queries may not be applicable in larger populations, search terms may not be closely related to illness severity |
| Feng & Shah (2022)[64] | Google search & Twitter (daily, April - May 2020) | COVID-19 | United States | The authors propose a multi lens framework for integrating open data sources over varying time frames for modelling emerging events, including Google search trends, Twitter trends and census population data. | The authors trained and evaluated regression models: Bayesian ridge regression, ridge regression, lasso LARS regression, random forest regression, Support Vector regression (SVR), linear regression, and k-neighbours regression, found merged data sources outperformed models with a single data source. | Data available in varying spatial units, internet data less precise due to imposed access limitations and privacy concerns, high levels of processing required for social media data |
| Gao *et al*  (2022)[58] | Baidu Search Index (BSI), TikTok index, Baidu information index (Daily; Jan - March 2020) | COVID-19 | China | The authors analysed BSI search data for 33 keywords from categories including name, symptoms, prevention, diagnosis, therapy and related diseases in addition to keywords (COVID-19, coronavirus, epidemic, infectious disease, pneumonia, SARS, influenza, vaccine) from Baidu information index and TikTok propose modelling that integrates multiple early warning tasks based on multisource Internet big data and combines multiple ensemble models. | The early warning model successfully found cryptic transmission before the traditional monitoring system, with models including internet search, social media and population mobility had the best predictive accuracy for cryptic transmission occurrence warning. With these models, the early warning signal was detected 13 days earlier than the official warnings of transmission. | Short study period (due to border closures), data limited to two provinces |
| Gong *et al* (2022)[54] | Baidu Index & Sina Micro Index (Daily; Jan - Mar 2020) | COVID-19 | China | Baidu Index and Sina Micro Index were used to determine correlation between COVID-19 cases and public attention in China using Time lag cross correlation, Spearman’s rank correlation to build predictive models utilising BDI and SMI with multiple linear regression models. | Using time lag cross correlation for new confirmed cases and new death cases, correlation varied, but was highest at lags between 3–16 days. In addition, BDI was more strongly correlated with case indicators than SMI. predicted values and actual values of the four models were 0.892, 0.762, 0.766, and 0.674, respectively. It shows that the four models perform well in predicting COVID-19 new cases. | Early keywords were ambiguous due to unknown nature of outbreak, limited to search engines, short study period |
| Gónzalez-Bandala *et al* (2020)[27] | Google Trends (Weekly; 2002-2019) | Respiratory Illnesses/ILI | Mexico | Pearson’s correlation Merge prediction and ARI forecast - using FFNN and projection model based on SoS. FFNN forecasting model was trained on seasonal ARI from 2008 - 1015 and forecast 2015 to 2016 season, merge prediction is composed by the forecasting model (FFNN), the projection model (SoS), and the endemic channels - | Using machine learning models with multivariate data had better RMSE, RMSPE, and MAPE and winter season forecasting using merged FFNN and SoS had a higher correlation coefficient compared with using FFNN or SoS individually. | Google correlate discontinued, limited spatial resolution country level only, study only covered four winter seasons |
| Guo *et al* (2017a)[35] | Baidu search (Daily; Jan 2011 - May 2015) | Influenza | China | The authors assessed and compared prediction performance of different penalized regression models using influenza incidence data and influenza search results | Overall, the ensemble elastic net regression model had the largest average AUC of 0.97. Internet search terms-based models able to capture peaks and troughs of seasonal influenza. | Large variety of Chinese language search keywords may be difficult to apply over large regions |
| Guo *et al* (2017b)[45] | Baidu search (Weekly; 2011-2014) | Dengue | China | The authors constructed dengue search index (DSI) from Baidu search query data to predict dengue, compared a range of machine learning models - support vector regression (SVR) model, step-down linear regression model, gradient boosted regression tree model (GBM), negative binomial regression model (NBM), least absolute shrinkage and selection operator (LASSO) linear regression algorithm and generalized additive model (GAM). Predictive performance and goodness of fit was assessed using RMSE and R-squared. | The SVR model has the best predictive performance RMSE values were consistently smallest for the 1-month-ahead predictions in 2014, across all regions with large R-squared values for Yunnan (0.976), Guangxi (0.970), Hunan (0.997), Fujian (0.981) and Zhejiang (0.985). | Data from rural areas is limited, model does not account for mosquito density and other external factors, model requires extensive processing time |
| Guo *et al*  (2021)[56] | Weibo & Google Flu Trends (Daily; Dec 2019 – March 2020) | COVID-19 | China | The authors compared four predictive algorithms using Weibo and Google trends to forecast new COVID-19 cases | In the four combination algorithms, GA&LR (WCT) had the best predictive R ​= 0.65 (p ​< 0.01), while the average test score of GCA&LSTM is the smallest at R ​= 0.43 (p ​< 0.01). | Limited to short term predictions, relevant keyword may vary over time as pandemic persists |
| Habibdoust *et al*  (2024)[67] | Google Trends  (Daily; 2020-2021) | COVID-19 | United States | Lagged daily search volumes for COVID symptoms over three time periods using GMDH-type neural network model to predict and forecast nonlinear data using 80% training and 20% testing periods to determine best fit. | Search terms “Fever,” “COVID Testing,” “Signs of COVID,” “COVID Treatment,” and ”Shortness of Breath” increased predictive accuracy of models, but results varied between study periods, where some models were more accurate at different times. Queries and case models increased predictive accuracy by 22.6%, 21%, and 37.3% improvement in NRMSE across the three periods. |  |
| Hassan Zadeh *et al*  (2019)[42] | Twitter  (Daily; 2013-2015) | Influenza | United States | The authors performed spatio-temporal big data analysis and visualization with GIS, to nowcast influenza outbreaks using Twitter data, point processes analyses to obtain temporal-spatial cross-correlations and mapped flu outbreaks with Twitter data to identify influenza hotspots. | The authors found clinical flu diagnosis lag 1 month behind online posts, monthly numbers of unique users posting about flu was a good measure of the number of patients with ILI symptoms with cross-correlation coefficient up to 0.90 between flu diagnosis and Twitter flu activity. Hot spots of twitter activity were identified using Moran’s spatial autocorrelations (p value <0.05), the top 10 regions were public non-residential places with shops and offices, and public entertainment areas. | Limited geolocation data, user defined data may not be valid, high levels of noise from social media |
| Ho *et al.*  (2018)[48] | Google Dengue Trends (Weekly; 2009 - 2014) | Dengue | Philippines | The authors explored temporal relationship between GDT and dengue incidence, using Pearson correlation, spatial patterns of GDT and dengue. Assessed search terms included dengue, signs and symptoms, treatment and prevention and mosquito for potential inclusion in disease surveillance. | GDT search terms including signs and symptoms were significantly associated with dengue (r = 0.394 to 0.747), terms were more relevant in some years than others. TSCC results revealed that GDT has a delayed effect (1–2 weeks) to the reported dengue incidence. Spatial association between cases and GDT was not significant in Manila. | Analysis of temporal and spatial relationship only, internet use patterns mean search trends may not be related to residence, low internet availability outside city means internet search may not be appropriate surveillance method |
| Husnayain *et al* (2019)[49] | Google Trends (Weekly; 2012-2016) | Dengue | Indonesia | Correlation analysis of dengue fever related search terms including disease definition, symptoms, treatment, and disease vector for early warning surveillance using Indonesian language search terms. | Google Trends data was strongly correlated with dengue, with R-value range from 0.921 to 0.937 (p ≤ 0.05, overall period) which showed increasing trend in epidemic periods (2015–2016). Google trends from a month prior were correlated with dengue (R-value≥0.7 and p ≤ 0.05) ranging from 0.755 to 0.773. | Temporal association analysis only, internet penetration increasing but comparatively lower |
| Kogan *et al* (2021)[69] | Google trends, UpToDate, ILINet, Twitter, Mobility data (Daily; Mar-Sept 2020) | COVID-19 | United States - Massachusetts (MA), New York (NY), and California (CA) | The authors evaluated multiple data sources as early warning signals for COVID-19 cases in selected US states Google search terms including "fever', "covid", "quarantine" and Twitter results for "covid", "corona", "epidemic", "flu", "influenza", "face mask", "spread", "virus", "infection", "fever", "panic buying", "state of emergency", "masks", "quarantine", "sars", and "2019-ncov") in addition to health data, mobility, and smart thermometer data; for event detection; time to event estimation, prediction and validation | Increases in digital data signals preceded confirmed cases and deaths by 2 to 3 weeks. Search trends for fever were correlated with increased temperature readings, while mobility changes associated with NPIs were indicative of downtrends ion cases. Multivariate time to event estimation found 50% of uptrends were predicted 2 weeks in advance, while 75% of uptrends were predicted 1 week in advance. | Spatial resolution – MUAP, state level only, smart device data may be limited outside the US. |
| Li *et al*  (2022)[57] | Baidu index, Microblogs (Daily; Dec 2019 - Jan 2020) | COVID-19 | China | Search results for "fever", "dry cough", "chest distress", "pneumonia", coronavirus" were used to develop forecasting models for comparison | Model 1 forecasting accuracy improved significantly compared with the baseline model using COVID-19 case counts only (model 1, t198=–1.732, P=.09). Baseline models were statistically significant (model 1, t198=–8.722, P<.001; model 2, t198=–5.000, P<.001, model 3, t198=–1.882, P=.06; model 4, t198=–4.644, P<.001; model 5, t198=–4.488, P<.001). Internet-based sources could provide a 2- to 6-day earlier warning for COVID-19 outbreaks | Early and short study period, did not utilise machine learning methods, symptom-based keywords won’t detect asymptomatic individuals |
| Li *et al*  (2023)[53] | Baidu index (Daily; Nov 2021- Mar 2022) | COVID-19 | China | Baidu search terms including prevention, symptoms, testing, treatment, vaccine, epidemic condition, and other frequent keywords were collected. Time series cross correlation was used to determine temporal association between cases and keywords. GAM was used to determine correlation between BI and numbers of infections. | Using GAM with Baidu search, the authors found the optimal early warning thresholds were P30 with a 7-day moving window size for Shanghai, an upper early warning threshold of P70 with a 7-day moving window size for Shenzhen, and an upper early warning threshold of P90 with a 14-day moving window size for Changchun. Baidu signals were used to detect signals of Omicron outbreaks in March 2022 - 40, 27, and 20 days in advance. | Short study period, limited number of outbreaks analysed, media coverages and preventative measures in place mean signal strength may be biased |
| Li *et al*  (2017)[47] | Baidu index (Weekly; 2011-2014) | Dengue | China | Time series cross correlation for Dengue cases, weather (rainfall and minimum temperature) with Dengue related Baidu search was performed for lag 1 to 16 weeks. GAM were applied to fit relationships between variables and Dengue cases | Baidu search for dengue with one-week lag showed a positive linear relationship with dengue cases, and the model including search terms (ICC:0.94 and RMSE:59.86) has a better predictive capability than the model without search terms (ICC:0.72 and RMSE:203.29). | Short model validation period (8 weeks), city specific study, media coverage bias |
| Liu *et al*  (2016)[44] | Baidu index (Weekly; 2010 - 2014) | Dengue | China | The authors used Baidu search to determine prediction threshold for dengue fever using, time series cross correlation and regression tree models. | The authors used CART models to identify threshold values for dengue outbreaks based on Baidu search changes. in Guangzhou mean incidence rate increased by 30-fold when search was ≥382 at lags of 1 to 3 weeks, while in Zhongshan mean incidence rate increased by 5.6-fold at lags of 1 – 5 weeks. | Digital data limited to areas with internet access, spatial resolution, and media coverage bias |
| Liu *et al*  (2019)[46] | Baidu index  (Monthly; 2011 – 2015) | Dengue | China | The authors compared GAM with GAMM (with autocorrelation component) including weather variables and Baidu search indexes to improve dengue prediction in Guangzhou city, | The predictive accuracy of the GAMM (R^2^: 0.95 and RMSE: 34.1) has a superior prediction capability compared with GAM (R^2^: 0.86 and RMSE: 121.9). | No comparison to univariate models, limited study period, temporal resolution of data low monthly vs weekly/daily |
| Lopreite *et al* (2021)[61] | Twitter (2018 - 2020 | COVID-19 | United Kingdom, Germany, France, Italy, Spain, Poland, and the Netherlands | The authors analysed Twitter data leading up to the pandemic to detect early notifications of COVID-19 pneumonia by comparing mentions of pneumonia related tweets | Analysis of pneumonia related tweets showed a significant increase in tweets mentioning pneumonia preceding official COVID-19 outbreak, anomalous increase in the number of symptom-based keywords during the weeks leading up to the peak in February 2020 in all countries except Germany | Focus on signal detection for outbreaks, difficult to detect unknown/unnamed outbreaks, pneumonia keyword prone to media coverage bias over time |
| Marques-Toledo *et al*  (2017)[50] | Twitter, Google Trends, Wikipedia (Daily; 2012- 2016) | Dengue | Brazil | Twitter mentions for "Dengue", "aedes" and "aegypti" were compared with Google search & Wikipedia article access logs for "Dengue" to explore spatial association and develop forecasting models using GAM at country and city level. | Dengue related tweets, GT and Wikipedia logs were strongly positively associated with Dengue cases (r = 0.87, p<0.001) (r = 0.92, p<0.001) and (r = 0.71, p<0,01) respectively at the country level. At the city level, tweets were useful for estimating and forecasting dengue, with 67% of cities had positive association with tweets and cases. Forecasting models including Tweets improved goodness of fit and were able to provide early warning up to 8 weeks in advance. | Limited population of Twitter users, prone to media coverage bias, spatial resolution may be limited |
| McClymont *et al* (2023)[68] | Google Trends, Google Mobility (Daily; Aug 2021-Nov 2021) | COVID-19 | Australia; Victoria | The authors used Google Search, Google Mobility, and weather factors to develop predictive models for COVID-19 cases and effective reproductive number (Reff) developing multivariate forecasting models with TSCC and ARIMA predictive models. | Evaluating a wide range of digital surveillance sources, the authors found mobility factors combined with weather improved model predictive accuracy. Transit station mobility and maximum temperature positively correlated from 2 days prior increasing up to 10 days, and 5-8 days prior for temperature. ARIMA model including both Tmax and TSM had the best fit and predictive accuracy (R^2^ = 0.948) compared with Tmax only (R^2^ = 0.943) or TSM only (R^2^ = 0.944) and cases only (R^2^ = 0.942). | Internet search queries biased by media coverage, broad search queries used, spatial resolution differences across variables (MUAP), short study period |
| Nagar *et al* (2014)[41] | Twitter & Google Trends (Daily; 2012 - 2013) | ILI | United States; New York City | Using Twitter and Google search keywords including "flu", "influenza", "gripe" and "high fever" to validate model predictive strength for ILI-ED visits, geographical surveillance to detect significant space-time disease clusters. | Twitter data was strongly correlated with ILI EC visits (R=.763), and outperformed daily GSQ data (R=.683). Comparing infection versus awareness, found infection-based tweets were better indicators of cases. For prediction of cases during the peak flu season, Predictive values for comparison of models found Tweet based model (MAPE=8.4) performed better than Google search model (MAPE=11.8). Using spatiotemporal analysis, the authors identified a primary cluster of a high ratio of high-probability sick tweeters to medium-probability infection tweeters in northern Brooklyn (RR=2.74; P<.00) from November through March. | Limited geolocation values, multiple languages in the region may impact keywords prevalence or usage, social media usage may not be representative of mobility or actual locations |
| Olukanmi *et al* (2021)[32] | Google Trends (Weekly; 2010 - 2018) | ILI | South Africa | Google trends (GT) data were used for ILI forecasting in South Africa comparing deep learning Long short-term memory (LSTM) and feedforward neural networks (FNN), machine learning Multiple linear regression (MLR), elastic net (EN), support vector machine (SVM), and statistical time series seasonal autoregressive integrated moving average (SARIMA) algorithms. | Predictive models using Google search data were used to forecast ILI incidence rates with accuracy close to ILI surveillance, of the ILI only, GT only and GT and ILI models, the GT and ILI models outperformed the other models. Deep learning model FNN had the best predictive performance with RMSE value=10.54 and MAE=7.33 followed by LSTM, SVR and MLR models. The authors found GT only data was able to predict ILI incidence rates but including both GT and ILI surveillance provided greater forecasting accuracy. | Prediction accuracy dropped as forecast horizon increased making it difficult to predict long term trends from search values, low spatial resolution available |
| Shen *et al*  (2020)[55] | Weibo (Daily; Nov 2019 - March 2020) | COVID-19 | China | Using COVID-19 related posts on Weibo, the authors used machine learning to identify illness related posts to determine association with cases and develop predictive models for Hubei province and the rest of mainland China. | Performance of machine learning models varied, with Random Forest models having the greatest precision, accuracy and recall for detecting COVID-19 related posts. Predictive models using reports of symptoms and diagnosis of COVID-19 were able to accurately forecast daily case counts 14 days ahead of official statistics, predictive power of sick posts on daily case counts were useful for both Hubei and non-Hubei regions, with varying effect sizes. | Results affected by media coverage bias, regional testing availability during outbreaks may impact results, retrospective data collection, users may overreport or underreport on social media |
| Sparks *et al* (2017)[39] | Twitter (Daily; July 2013 - Sept 2014) | Influenza | Australia | Tweets including symptoms keywords including cough, fever, headache were collected from Australian geotagged sources to explore the temporal changes and seasonal influences over time points. Poisson regression models were used to fit the forecast models using the cleaned training data including symptoms keywords (cough, fever, headache) collected from Australian geotagged sources | Daily keywords counts were able to provide timely information in real-time on emerging flu outbreaks and increases compared with conventional surveillance over two flu seasons in 2013 and 2014. | Difficult to classify and determine appropriate keywords, noise from external sources and unrelated usage, limited spatial resolution or number of users with geotagging active |
| Stolerman *et al* (2023)[65] | Google Trends, Twitter, UpToDate, Apple Mobility (Daily, Jan 2020 - Jan 2022) | COVID-19 | United States | Using machine learning to identify early warning signals, data was collected from a range of digital sources, to develop an early warning system using digital proxies and evaluate thresholds for early, missed, or late warnings using Google Trends, UpToDate, Twitter API and Apple Mobility with spatial clusters | The lag between internet search signals may be in the range of 4 to 6 weeks preceding an outbreak, or an early warning signal. The models with multiple sources successfully identified most outbreaks during this period with early warning and soft warning thresholds for events. The model was able to detect events occurring at the county level and performed better than the case only early warning with fewer false alarms. | Google search trends lost power over time due to media coverage bias, study generalizability may be lower due to low data availability in some counties and data not available outside the US |
| Tu *et al*  (2021)[52] | Baidu index  (Daily; Jan 2020 - April 2020) | COVID-19 | China | Baidu search index values for common COVID-19 symptoms (cough, fever, fatigue) were compared with cases using Spearman's rank correlation, regional distributions were analysed | Daily growth of confirmed cases and Baidu index values for each COVID-19-related symptoms showed positive correlations during the outbreak (fever: *r*_s_=0.705; cough: *r*_s_=0.592; fatigue: *r*_s_=0.629; sputum production: *r*_s_=0.648; shortness of breath: *r*_s_=0.656). The average search-to-confirmed interval was 19.8 days while optimal time lags for Baidu Index were 4 days for cough, 2 days for fatigue, 3 days for sputum production, 1 day for shortness of breath, and 0 days for fever. Baidu searches peaked prior to the peak of the outbreak. | Limited to one data source, keywords may lose accuracy or change usage over time, short study period |
| Yang *et al*  (2023)[37] | Baidu Index (Weekly; 2012 - 2019) | ILI | China | The authors compared performance of machine learning models (Random Forest and XGBoost) with deep learning models (LSTM and GRU) with multiattention deep-learning model based on LSTM (MAL) using Baidu index, demographic data, and weather variables to develop to predict ILI% and ILI% positive%. | When comparing multiple models and inclusion of a wide range of variables, the authors found models with ILI%, climate, demography, and Baidu search index had the best prediction effect, with the explained variance score reaching 0.78, R^2^ reaching 0.76, MAE of 0.08, and MSE of 0.01 | Clinical surveillance data may vary over time, web search keywords may not represent healthcare seeking |
| Yang *et al*  (2021)[29] | Google Trends, GFT (Weekly; 2014-2020) | ILI | United States | ARGOX (Augmented Regression with Google Data Cross Space) modelling was used for real time tracking of IILI epidemics in the US, combining internet search data with traditional surveillance data to determine spatial correlation structure and changing internet search patterns. | ARGOX model with GT had 28% error reduction in MSE, 15 % error reduction in MAE compared to naïve estimates without GT. Model performance was consistent over seasons, with at least 15% error reduction in MSE compared to the best alternative method in every season from 2014 to 2019. | Results rely on consistency of search index, media coverage bias, ILI% also prone to noise due to limited sample size |
| Yang *et al*  (2023)[66] | Google Trends, Twitter (Daily; Jan 2020 – March 2020) | COVID-19 | Canada | The authors used time Google Search Trends and Twitter data (symptoms including cough, runny nose, anosmia, sore throat etc.) to predict COVID-19, performed time-lagged cross-correlation analyses using denoised signals and LSTM to predict COVID-19 cases | Using TSCC, Google and Twitter symptom -related search terms were strongly correlated with time lag 1-13 days. Best fit predictive LSTM models used Google search terms (MSE = 124.78, R2 = 0.88, adjusted R2 = 0.87), although Twitter also improved predictive accuracy of the models. | Short study period, results may be biased by media coverage, models prone to over-fitting, selected keywords may lack generalisability |
| Yom-Tov *et al* (2022)[60] | Bing search (Weekly; March 2020 - Dec 2021) | COVID-19 | England | Search queries from Bing search attributed to unique users were aggregated by geolocation, this information was used to detect spatial variations in search queries for COVID symptoms related terms and how these related to outbreak detection | During the initial waves of the pandemic, Bing searches for “fever” and “cough” were strongly associated with future case counts, preceding rises by up to 21 days. Increases in search patterns were predictive of rises in future case counts within a week, reaching a peak Area Under Curve of 0.82 during the initial phase of the pandemic, this decreased over time. | Search queries and keywords less useful over time with increased media coverage and changing symptom presentation, Bing search potentially less representative of a population with lower usage |
| Yousefinaghani *et al*  (2021)[63] | Twitter, Google Trends (Weekly; Jan 2020 - Sept 2020) | COVID-19 | United States & Canada | Interest in COVID related keywords from Twitter and Google Trends were used to visualise trends over time in relation to COVID-19 cases, and to detect pandemic waves using anomaly detection over time and determine most predictive symptoms for pandemic trends. | IN Canada, up to 83% of initial waves were detected 1 week in advance using Google symptom searches, while in the US, 78% of first outbreaks were detected 1-2 weeks in advance based on Tweets. The most useful keywords for detecting emerging outbreaks were “fever” and “cough,” though association declined as pandemic continued. | Keywords and usage affected by media coverage; limited forecasting due to short study period |
| Yousefinaghani *et al*  (2019)[40] | Twitter (Daily; 2017 - 2018) | Avian influenza | Global | Twitter data was used to evaluate use in Avian influenza for the detecting the onset of outbreaks and determining event magnitude using anomaly detecting algorithm Seasonal-Hybrid Extreme Studentized Deviate (SH-ESD). | Up to 75% of real-world outbreak notifications of avian influenza were identifiable from Twitter, and as much as one-third of outbreak notifications were reported on Twitter earlier than official reports. | Irrelevant or unrelated Tweets contribute to noise, reported cases in animals rather than humans |
| Zhang *et al*  (2018)[30] | Google Trends (Weekly; 2011 - 2016) | Influenza | Australia; Queensland | Climate data and search trends related to influenza were used to explore temporal correlation with influenza and explore predictive models for seasonal outbreaks of influenza using SARIMA modelling and Regression Tree analysis. | Influenza infections were significantly corrected with GT (influenza)at lag of 1–7 weeks and temperature at lag of 1–10 weeks in Brisbane and Gold Coast. SARIMA models with GT and temperature data had better predictive performance. Mean influenza notifications increased by 5.3-fold in Brisbane when AR for flu at 1 week lag was ≥1168, and by 5-fold in Gold Coast when 1 week lag AR was ≥187. | Single search term “influenza” may have reduced accuracy of models, data only available at state level, may be affected by media coverage bias |
| Zhang *et al*  (2019)[38] | Google Trends, Baidu Index (Weekly; 2010-2018) | Influenza | Australia, China, US & UK | The correlation between Baidu and Google search terms (influenza) and influenza notifications in China, Australia, UK, and the US, using TSCC and SARIMA model to determine seasonal patterns of influenza and explore temporal relationships between search terms and outbreaks | The inclusion of search metrics improved the performance of the SARIMA model (China = 0.96, US = 0.97, UK = 0.96, p < 0.01) and low MAPE values (China = 16.76, US = 96.97, UK = 125.42). There were significant associations between local internet search data and influenza surveillance data at 1–7 weeks lag. | Media coverage bias, limited to one search term “influenza” rather than “flu,” only laboratory confirmed cases included |
| Zhou *et al*  (2018)[33] | Google Trends (Daily, 2004 - 2015) | Influenza | Global | Using influenza case data, with influenza-related Google query data, influenza news data, international air transportation data integrated in a Multivariate Hidden Markov Model (MHMM), to estimate and predict influenza transmission globally. | Using a range of digital surveillance sources, the proposed method reached 90.26 to 97.10% real-time detection accuracy over the 2005-2015 study period for global influenza epidemics. For detecting unmonitored epidemics, this study detected influenza epidemics from imported cases on air transportation, successfully predicting an influenza epidemic before it occurs with average 89.20% accuracy. Multivariate model was more sensitive and accurate at detecting epidemics compared with naïve models. | Outbreak detection only, no forecasting or predictive, limited clinical surveillance in some countries make models harder to fit, morbidity data may be delayed or missing so harder to use for timely outbreak detection |
| Zimmer *et al* (2018)[28] | Wikipedia (Weekly; 2008-2016) | ILI | United States | The authors developed a predictive framework for influenza transmission based on Wikipedia search data using a SIRS model with humidity and daily reproductive number. | With weekly Wikipedia data, the model improved compared with case only models from CDC ILI, daily Wikipedia data improved model predictive ability in most seasons, with an average gain of 21%. The best predictive capacity was over the 1- and 2-week periods, with accuracy decreasing at 4 weeks. | Wikipedia search intensity may vary over a season, affected by media coverage bias, does not distinguish unique users or with precise geolocations. |
